# CCR5Δ32 and HLA allele diversity in bone marrow donors from southern Brazil

**DOI:** 10.1590/1678-4685-GMB-2023-0198

**Published:** 2024-07-29

**Authors:** Bruna Kulmann-Leal, Joel Henrique Ellwanger, Ana Cristina Arend, Luiz Fernando Job Jobim, Mariana Jobim, Rafael Tomoya Michita, Sidia Maria Callegari-Jacques, Luís Cristóvão de Moraes Sobrino Pôrto, José Artur Bogo Chies

**Affiliations:** 1 Universidade Federal do Rio Grande do Sul (UFRGS), Departamento de Genética, Laboratório de Imunobiologia e Imunogenética, Porto Alegre, RS, Brazil.; 2 Universidade Federal do Rio Grande do Sul (UFRGS), Departamento de Genética, Programa de Pós-Graduação em Genética e Biologia Molecular, Porto Alegre, RS, Brazil.; 3 Hospital de Clínicas de Porto Alegre (HCPA), Unidade de Imunologia de Transplantes e Medicina Personalizada, Porto Alegre, RS, Brazil.; 4 Baylor College of Medicine, Section of Infectious Diseases, Department of Medicine, Houston, Texas, USA.; 5 Universidade Federal do Rio Grande do Sul (UFRGS), Departamento de Estatística, Porto Alegre, RS, Brazil.; 6 Universidade do Estado do Rio de Janeiro (UERJ), Laboratório de Histocompatibilidade e Criopreservação, Rio de Janeiro, RJ, Brazil.

**Keywords:** CCR5Δ32, immunogenetics, HIV, HLA, viral suppression

## Abstract

Transplantation of stem cells derived from donors with CCR5Δ32 homozygous genotype is a potential strategy to achieve both the control of malignant hematological disease as well as sustained remission of the HIV infection, and researchers in different countries are looking for CCR5Δ32 homozygous donors to replicate such a ‘double-target’ strategy. We determined the frequency of the CCR5Δ32 variant in a sample of 1,398 bone marrow donors from Rio Grande do Sul State, Brazil. This study also evaluated whether *HLA-A*, *HLA-B* and *HLA-DRB1* genotypes are homogeneously distributed between CCR5Δ32 carriers and non-carriers in a population characterized by a significant genetic admixture. The CCR5Δ32 allele frequency was 7.4% (CI_0.95_ 6.4-8.4%), and the frequency of the Δ32/Δ32 homozygous genotype was 0.72% (CI_0.95_ 0.34-1.31%). In general, HLA genotypes are homogeneously distributed between CCR5Δ32 carriers and non-carriers. Considering the large number of bone marrow donors in Brazil and the high CCR5Δ32 allele frequency observed in our study, our results clearly indicate the existence of a considerable amount of potential CCR5Δ32 homozygous bone marrow donors in southern Brazil, suggesting that an active search for these donors is not only feasible but an attractive and promising strategy towards effective HIV infection control and treatment.

## Introduction

Despite intense research involving HIV/AIDS since the 1980s, there is still no effective cure for HIV infection. Scientific efforts are still extremely necessary to reduce HIV-associated conditions, including AIDS and the chronic inflammation observed in patients on antiretroviral therapy (ART). In the clinical course of HIV infection, there is a large depletion of CD4+ T cells since the CD4 molecule is the main binding receptor which HIV uses to penetrate cells. The HIV gp120 surface protein interacts with CD4 protein. After this initial gp120/CD4 binding, the gp120 is displaced, leaving the HIV gp41 protein free, which binds to a host cellular co-receptor, more frequently CCR5, a chemokine receptor involved in inflammatory reactions. In this sense, CCR5 is an accessory molecule needed for effective HIV infection. Because of its direct role in this pathogenic process, multiple aspects of CCR5 have been studied in the context of HIV infection and AIDS progression (Gottlieb et al., 1981; Broder and Gallo, 1984; Montagnier et al., 1984; Moore et al., 1990; Berson et al., 1996; Alkhatib, 2009).

In 1996 a 32 base-pair deletion in the *CCR5* gene was described and subsequently named as CCR5Δ32 (Liu et al., 1996). The CCR5Δ32 variant consists of a frameshift mutation in the coding region of the *CCR5* gene (exon 3), which generates a premature stop codon and the consequent expression of a non-functional protein, which is not transported to the cell surface, thus preventing its function as chemokine receptor. Considering that individuals who show this variant in homozygosity do not express the CCR5 molecule in the surface of their cells, CCR5-tropic HIV-1 strains are prevented from successfully interacting with host cells, blocking the process of viral penetration (Dean et al., 1996; Liu *et al.*, 1996; Samson et al., 1996; Proudfoot, 2002; Venkatesan et al., 2002; Ellwanger et al., 2020 a ,b). Nevertheless, some HIV strains can interact with other accessory molecules (especially CXCR4) (Hoffmann, 2007). Therefore, although CCR5Δ32 homozygosis is a natural factor associated with HIV infection resistance, this condition *per se* is not capable to abrogate infection of non CCR5-tropic strains.

The CCR5Δ32 allele has a heterogeneous distribution in populations around the world. This variant has a European origin and is practically absent in African, Asian, and Amerindian populations (Martinson et al., 1997; Libert et al., 1998; Solloch et al., 2017). The Brazilian population is highly admixed, with an important genetic contribution of African, Native-American, and European populations (Kulmann-Leal et al., 2021). The presence of Euro-descendant individuals is greater in the southern region of Brazil, which reflects the higher frequencies of the CCR5Δ32 allele in this region within the Brazilian territory. The CCR5Δ32 allele frequency is around 4-6% in Brazil, but it can be as high as 9% in specific southern populations (as observed in a study performed in Paraná State with Euro-descendant population) (Boldt et al., 2009; Silva-Carvalho et al., 2016; Kulmann-Leal *et al.*, 2021). For example, based on studies performed with samples of healthy population ranging from *n*=103 to *n*=334 individuals, in Rio Grande do Sul State the CCR5Δ32 allele is commonly observed at frequencies between 6% to 7% (Vargas et al., 2006; Ellwanger et al., 2018; Ellwanger *et al.*, 2020c).

Since its discovery, the CCR5Δ32 variant has been subject of numerous studies in the context of HIV infection. In 2009, Hütter et al. (2009) published an innovative and striking study about the ‘Berlin Patient’, a 40-year-old HIV-infected man who was diagnosed with acute myeloid leukemia. As a treatment for the neoplasia, the patient underwent an allogeneic stem cell transplant from an HLA compatible donor, but also homozygous for the CCR5Δ32 allele. The donor was chosen in order to carry out a possible combined (‘double-target’) therapy for leukemia and HIV infection, since the donor’s cells without CCR5 expression could not be infected by the patient’s CCR5-tropic HIV strains. After the transplant, the patient no longer received ART and his viral load was followed for the next 20 months. Remarkably, active and replicating viral particles were not detected in that period. Three years after the procedure, Hütter and Thiel (2011) updated the Berlin Patient’s clinical situation, reporting the continuous absence of replicative viral particles still without ART need (Hütter *et al.*, 2009; Hütter and Thiel, 2011). The Berlin Patient eventually died in 2020 due to a relapse of the leukemia, although he was still HIV-free (Kulmann-Leal et al., 2021).

Gupta et al. (2019) reported a second successful case of sustained remission of HIV infection. Following a similar protocol described by Hütter et al. (2009), a patient diagnosed with HIV-1 infection and IVb stage Hodgkin’s lymphoma underwent an allogeneic stem cell transplant with CCR5Δ32 homozygous cells. ART was discontinued at day 510, and in the same way of the first reported case, the presence of HIV was no longer observed after the transplant. Subsequently, Gupta *et al.* (2020) reinforced the success of the procedure 30 months after the transplant, describing more details regarding the case. This second individual to achieve sustained remission of HIV infection is known as the ‘London Patient’ (Gupta *et al.*, 2019; 2020). Recently, another case of HIV sustainable remission after transplant with CCR5Δ32 homozygous cells was announced, the ‘Düsseldorf patient’ (Jensen et al., 2023).

Considering the possibility of obtaining a sustained remission of HIV infection after stem cell transplant, different research groups are investigating the feasibility of this procedure in various populations. Solloch et al. (2017) summarized CCR5Δ32 variant frequencies for 87 countries, also describing the CCR5Δ32 genotypes for over 1.3 million individuals from Germany, Poland, and UK. Genotype data was obtained from potential hematopoietic stem cell donors from the German Bone Marrow Donor Center (DKMS). Solloch *et al.* (2017) estimated that a German HIV+ individual who needs a stem cell transplant has a 28.7% probability of finding a donor both HLA compatible and CCR5Δ32 homozygous. Also, Enrich et al. (2018) genotyped 20,236 cord blood units (CBUs) from The Spanish Bone Marrow Registry (REDMO), and found a total of 130 CBUs homozygous for the CCR5Δ32 variant. Both studies show that, although the CCR5Δ32 allele is not highly prevalent in most countries, it is possible to find CCR5Δ32 homozygous individuals in hematopoietic stem cell donor registries that could contribute with new attempts of sustained HIV suppression in HIV+ individuals who eventually need a stem cell transplant due to malignant hematological diseases (Solloch *et al.*, 2017; Enrich *et al.*, 2018).

The search for CCR5Δ32 homozygous individuals as potential donors to HIV+ patients in bone marrow transplants due to malignant hematological diseases represents a cutting-edge initiative in HIV/AIDS research. In Brazil, the National Registry of Bone Marrow Volunteer Donors (*Registro Nacional de Doadores de Medula Óssea* - REDOME) is responsible for gathering information from people willing to donate bone marrow to anyone who needs a transplant, having more than 5 million registered members. In this context, the main objective of this study was to answer this question: (I) what is the CCR5Δ32 allele frequency in bone marrow donors from southern Brazil? Also, considering a potential association between HLA alleles and chemokine system genes (Favorova et al., 2002; Öckinger et al., 2010; Javor et al., 2015), the secondary objective of this study was to answer this exploratory question: (II) are the HLA genotypes homogeneously distributed between CCR5Δ32 carries and non-carries? The potential clinical implications of this research include benefits for HIV+ individuals who eventually need a stem cell transplant due to malignant hematological diseases, strengthening the proof-of-concept described by Hütter et al. (2009) and Gupta et al. (2019).

## Material and Methods

### DNA samples and ethical aspects

The genomic DNA samples used in the study come from bone marrow donors and were provided by the *Unidade de Imunologia de Transplantes e Medicina Personalizada* (Transplant Immunology and Personalized Medicine Unit) of *Hospital de Clínicas de Porto Alegre* (HCPA, Porto Alegre, Brazil), which is responsible for REDOME in Porto Alegre, the capital of Rio Grande do Sul, the southernmost state of Brazil. HLA typing was performed in the Transplant Immunology and Personalized Medicine Unit of HCPA using the LABType SSO Typing Test methodology (One Lambda, CA, USA) for loci A, B and DRB1. Of note, the population of Rio Grande do Sul State is composed mostly of Euro-descendant individuals (Pena et al., 2011). A total of 1,398 blood samples were investigated in this study, of whom *HLA-A*, *HLA-B* and *HLA-DRB1* alleles were determined previously. Only individuals recently registered as bone marrow donors (between 2019 to 2020) were included in the study. All potential donors signed a consent form allowing the genetic analysis of tissue compatibility antigens for transplantation purposes, in which *CCR5* genotyping is included. This study was approved by the research ethics committees of *Hospital de Clínicas de Porto Alegre* and *Universidade Federal do Rio Grande do Sul* (# 20460719.6.0000.5327).

### CCR5Δ32 genotyping

The CCR5Δ32 variant (rs333) was genotyped according to Chies and Hutz (2003) and minor adaptations by Ellwanger et al. (2020c). In brief, the fragments of interest were amplified by conventional PCR and the primers used in the reaction were: CCR5a 5’-GGTCTTCATTACACCTGC-3’; CCR5b 5’-AGGATTCCCGAGTAGCAGATG-3’. Genotyping was performed by analyzing the amplicons on a 3% agarose gel under UV light. A single 137 base-pair band represents the wild-type homozygous genotype; a 137 base-pair band associated with a 105 base-pair band indicates heterozygous genotype; a single 105 base-pair band indicates the variant homozygous genotype.

### Statistical analysis

CCR5Δ32 allele and genotype frequencies were calculated, and respective Fisher’s exact 95% confidence intervals (C.I.) were obtained using WinPEPI version 11.65 (Abramson, 2011). Hardy-Weinberg equilibrium was evaluated using the chi-square goodness of fit test. Due to the low frequency of CCR5Δ32 homozygous genotypes, the frequencies of ‘CCR5Δ32 carriers’ (CCR5Δ32 homozygous individuals grouped with heterozygous individuals) and ‘CCR5Δ32 non-carriers’ (individuals with wild-type homozygous genotype) were also calculated. The total number of individuals (*n*=1,398) was included in these initial analyses used to answer our first research question.

We subsequently assessed whether HLA genotypes were homogeneously distributed between CCRΔ32 carriers and non-carriers (our second research question). For this, *HLA-A*, *HLA-B* and *HLA-DRB1* genotype and allele frequencies were compared between CCRΔ32 carriers and non-carriers using Fisher’s exact tests. *P*-values <0.05 were considered statistically significant. We used the software GENEPOP (available at: https://genepop.curtin.edu.au/) (Raymond and Rousset, 1995; Rousset, 2008) to estimate *HLA* allele and genotype frequencies, test for Hardy-Weinberg equilibrium, and to compare allele and genotype frequencies between groups. 

## Results

### CCR5Δ32 genotype and allele frequencies

Table 1 shows the CCR5Δ32 genotype and allele frequencies among bone marrow donors genotyped in this study (*n*=1,398). Most donors (86%) do not carry the CCR5Δ32 allele. We observed a CCR5Δ32 allele frequency of 7.4% (CI_0.95_ 6.4 - 8.4%), which is higher than the average Brazilian frequency of 4-6% (Kulmann-Leal et al., 2021). In total, ten Δ32/Δ32 homozygous individuals were found (0.72%). Genotypes were distributed according to the expected for the Hardy-Weinberg equilibrium (*p*=0.345). Some samples showed uncertain HLA genotypes (*n*=208) and were excluded from the analyses onwards.

**Table 1 -:** CCR5Δ32 genotype and allele frequencies (*n* and %) among bone marrow donors.

CCR5Δ32	Bone marrow donors (n=1,398)	95% Confidence Interval (C.I.)
wt/wt	1202 (85.98%)	84.05% - 87.76%
wt/Δ32	186 (13.30%)	11.57% - 15.20%
Δ32/Δ32	10 (0.72%)	0.34% - 1.31%
Δ32 carrier	196 (14.02%)	12.24% - 15.95%
Δ32 non-carrier	1202 (85.98%)	84.05% - 87.76%
Δ32 allele frequency	0.074	0.064 - 0.084

*n*, number of individuals. wt/wt, wild homozygous genotype. wt/Δ32, heterozygous genotype, Δ32/Δ32, variant homozygous genotype.

### Distribution of CCR5Δ32 alleles in CCR5Δ32 carriers and non-carriers

Table 2 shows the allele frequencies of the three HLA loci investigated in the study (*n*=1,190). Nineteen *HLA-A*, 30 *HLA-B*, and 13 *HLA-DRB1* alleles were found in blood samples of bone marrow donors. The three most frequent alleles of each HLA loci were: HLA-A*2 (28.24%), HLA-A*24 (11.09%), and HLA-A*3 (9.71%); HLA-B*44 (12.44%), HLA-B*35 (11.09%), and HLA-B*15 (8.66%); HLA-DRB1*7 (14.41%), HLA-DRB1*11 (13.28%), and HLA-DRB1*13 (12.77%) (Table 2). 

**Table 2 -:** *HLA-A* , *HLA-B* and *HLA-DRB1* allele frequencies in 1,190 bone marrow donors.

*HLA-A*			*HLA-B*			*HLA-DRB1*		
Allele	(number of alleles)	Frequency (%)	Allele	(number of alleles)	Frequency (%)	Allele	(number of alleles)	Frequency (%)
HLA-A*2	672	28.24	HLA-B*44	296	12.44	HLA-DRB1*7	343	14.41
HLA-A*24	264	11.09	HLA-B*35	264	11.09	HLA-DRB1*11	316	13.28
HLA-A*3	231	9.71	HLA-B*15	206	8.66	HLA-DRB1*13	304	12.77
HLA-A*1	229	9.62	HLA-B*7	204	8.57	HLA-DRB1*4	282	11.85
HLA-A*29	132	5.55	HLA-B*51	198	8.32	HLA-DRB1*15	244	10.25
HLA-A*68	127	5.34	HLA-B*8	133	5.59	HLA-DRB1*1	241	10.13
HLA-A*11	122	5.13	HLA-B*40	130	5.46	HLA-DRB1*3	223	9.37
*HLA-A* *32	101	4.24	HLA-B*14	128	5.38	HLA-DRB1*8	129	5.42
HLA-A*31	98	4.12	HLA-B*18	126	5.29	HLA-DRB1*14	110	4.62
HLA-A*23	97	4.08	HLA-B*38	70	2.94	HLA-DRB1*16	80	3.36
HLA-A*33	78	3.28	HLA-B*27	66	2.77	HLA-DRB1*9	48	2.02
HLA-A*30	75	3.15	HLA-B*13	65	2.73	HLA-DRB1*10	31	1.30
HLA-A*26	72	3.03	HLA-B*49	61	2.56	HLA-DRB1*12	29	1.22
HLA-A*25	33	1.39	HLA-B*57	60	2.52			
HLA-A*66	16	0.67	HLA-B*50	49	2.06			
HLA-A*74	16	0.67	HLA-B*39	43	1.81			
HLA-A*34	8	0.34	HLA-B*58	43	1.81			
HLA-A*36	7	0.29	HLA-B*45	33	1.39			
HLA-A*69	2	0.08	HLA-B*52	29	1.22			
			HLA-B*53	28	1.18			
			HLA-B*55	27	1.13			
			HLA-B*41	27	1.13			
			HLA-B*48	27	1.13			
			HLA-B*37	24	1.01			
			HLA-B*42	15	0.63			
			HLA-B*56	12	0.50			
			HLA-B*47	10	0.42			
			HLA-B*81	4	0.17			
			HLA-B*46	1	0.04			
			HLA-B*73	1	0.04			

The total number of alleles is twice the number of individuals analyzed.

Alleles were distributed according to the expected for the Hardy-Weinberg equilibrium for *HLA-A* and *HLA-DRB1* (*p*=0.093 and *p*=0.187, respectively). On the other hand, the *HLA-B* locus showed a deviation from the Hardy-Weinberg equilibrium (*p*=0.024). However, this result may be explained by the large number of tests carried out. Table 3 details the *HLA* allele frequencies among CCR5Δ32 carriers (*n*=174) and non-carriers (*n*=1,016), and Table 4 shows the results of the statistical comparison of the allele frequencies of the three *HLA* loci evaluated here (*HLA-A*, *HLA-B* and *HLA-DRB1*) between CCR5Δ32 carriers and non-carriers. No test showed statistical significance (*p*>0.05), indicating a homogeneous distribution of *HLA* alleles among CCR5Δ32 carriers and non-carriers (Table 4).

**Table 3 -:** *HLA-A* , *HLA-B* and *HLA-DRB1* allele frequencies in 174 ‘CCR5Δ32 carriers’ and 1,016 ‘CCR5Δ32 non-carriers’ bone marrow donors.

CCR5Δ32 carriers (n=174)								
*HLA-A*			*HLA-B*			*HLA-DRB1*		
Allele	(number of alleles)	Frequency (%)	Allele	(number of alleles)	Frequency (%)	Allele	(number of alleles)	Frequency (%)
HLA-A*2	103	29.60	HLA-B*44	27	7.76	HLA-DRB1*7	45	12.93
HLA-A*24	34	9.77	HLA-B*35	39	11.21	HLA-DRB1*11	50	14.37
HLA-A*3	27	7.76	HLA-B*15	40	11.49	HLA-DRB1*13	46	13.22
HLA-A*1	40	11.49	HLA-B*7	29	8.33	HLA-DRB1*4	42	12.07
HLA-A*29	18	5.17	HLA-B*51	33	9.48	HLA-DRB1*15	36	10.34
HLA-A*68	20	5.75	HLA-B*8	19	5.46	HLA-DRB1*1	39	11.21
HLA-A*11	16	4.60	HLA-B*40	11	3.16	HLA-DRB1*3	33	9.48
*HLA-A* *32	23	6.61	HLA-B*14	17	4.89	HLA-DRB1*8	22	6.32
HLA-A*31	10	2.87	HLA-B*18	19	5.46	HLA-DRB1*14	11	3.16
HLA-A*23	11	3.16	HLA-B*38	13	3.74	HLA-DRB1*16	14	4.02
HLA-A*33	13	3.74	HLA-B*27	14	4.02	HLA-DRB1*9	4	1.15
HLA-A*30	10	2.87	HLA-B*13	11	3.16	HLA-DRB1*10	4	1.15
HLA-A*26	10	2.87	HLA-B*49	9	2.59	HLA-DRB1*12	2	0.57
HLA-A*25	7	2.01	HLA-B*57	9	2.59			
HLA-A*66	2	0.57	HLA-B*50	12	3.45			
HLA-A*74	3	0.86	HLA-B*39	9	2.59			
HLA-A*34	0	0	HLA-B*58	4	1.15			
HLA-A*36	1	0.29	HLA-B*45	5	1.44			
HLA-A*69	0	0	HLA-B*52	3	0.86			
			HLA-B*53	5	1.44			
			HLA-B*55	4	1.15			
			HLA-B*41	6	1.72			
			HLA-B*48	2	0.57			
			HLA-B*37	4	1.15			
			HLA-B*42	2	0.57			
			HLA-B*56	1	0.29			
			HLA-B*47	1	0.29			
			HLA-B*81	0	0			
			HLA-B*46	0	0			
			HLA-B*73	0	0			
**CCR5Δ32 non-carriers (n=1,016)**								
***HLA-A***			***HLA-B***			***HLA-DRB1***		
Allele	(number of alleles)	Frequency (%)	Allele	(number of alleles)	Frequency (%)	Allele	(number of alleles)	Frequency (%)
HLA-A*2	569	28.00	HLA-B*44	269	13.24	HLA-DRB1*7	298	14.67
HLA-A*24	230	11.32	HLA-B*35	225	11.07	HLA-DRB1*11	266	13.09
HLA-A*3	204	10.04	HLA-B*7	175	8.61	HLA-DRB1*13	258	12.70
HLA-A*1	189	9.30	HLA-B*15	166	8.17	HLA-DRB1*4	240	11.81
HLA-A*29	114	5.61	HLA-B*51	165	8.12	HLA-DRB1*15	208	10.24
HLA-A*68	107	5.27	HLA-B*40	119	5.86	HLA-DRB1*1	202	9.94
HLA-A*11	106	5.22	HLA-B*8	114	5.61	HLA-DRB1*3	190	9.35
*HLA-A* *31	88	4.33	HLA-B*14	111	5.46	HLA-DRB1*8	107	5.27
HLA-A*23	86	4.23	HLA-B*18	107	5.27	HLA-DRB1*14	99	4.87
HLA-A*32	78	3.84	HLA-B*38	57	2.81	HLA-DRB1*16	66	3.25
HLA-A*33	65	3.20	HLA-B*13	54	2.66	HLA-DRB1*9	44	2.17
HLA-A*30	65	3.20	HLA-B*27	52	2.56	HLA-DRB1*10	27	1.33
HLA-A*26	62	3.05	HLA-B*49	52	2.59	HLA-DRB1*12	27	1.33
HLA-A*25	26	1.28	HLA-B*57	51	2.51			
HLA-A*66	14	0.69	HLA-B*58	39	1.92			
HLA-A*74	13	0.64	HLA-B*50	37	1.82			
HLA-A*34	8	0.39	HLA-B*39	34	1.67			
HLA-A*36	6	0.30	HLA-B*45	28	1.38			
HLA-A*69	2	0.10	HLA-B*52	26	1.28			
			HLA-B*48	25	1.23			
			HLA-B*53	23	1.13			
			HLA-B*55	23	1.13			
			HLA-B*41	21	1.03			
			HLA-B*37	20	0.98			
			HLA-B*42	13	0.64			
			HLA-B*56	11	0.54			
			HLA-B*47	9	0.44			
			HLA-B*81	4	0.20			
			HLA-B*46	1	0.05			
			HLA-B*73	1	0.05			

The total number of alleles is twice the number of individuals analyzed.

**Table 4 -:** Comparison of allele frequencies of *HLA-A*, *HLA-B* and *HLA-DRB1* loci between CCR5Δ32 carriers and non-carriers.

Locus	p-value^1^
*HLA-A*	0.598
*HLA-B*	0.408
*HLA-DRB1*	0.783

^1^ Fisher’s exact test.

### Distribution of CCR5Δ32 genotypes in CCR5Δ32 carriers and non-carriers

Due to the large number of genotypes and consequent small number of individuals in each class, detailed frequencies of *HLA-A*, *HLA-B*, and *HLA-DRB1* genotypes are detailed in Tables S1, S2 and S3. Regarding *HLA* genotypes, all loci were distributed according to the expected for the Hardy-Weinberg equilibrium (*p*>0.05 in all tests). Table 5 shows the results of the comparison of the genotype frequencies of the *HLA* loci observed between CCR5Δ32 carriers and non-carriers. As observed in the tests involving alleles, no result showed statistical significance (*p*>0.05), indicating a homogeneous distribution of *HLA* genotypes among CCR5Δ32 carriers and non-carriers (Table 5).

**Table 5 -:** Comparison of genotype frequencies of *HLA-A*, *HLA-B* and *HLA-DRB1* loci between CCR5Δ32 carriers and non-carriers.

Locus	p-value^1^
*HLA-A*	0.614
*HLA-B*	0.381
*HLA-DRB1*	0.782

^1^ Fisher’s exact test.

## Discussion

The works of Hütter et al. (2009), Gupta et al. (2019), and Jensen et al. (2023) were pioneers in achieving sustained remission of HIV infection without the need for continuous use of ART. By deliberately looking for donors of hematopoietic stem cells who were also homozygous to the CCR5Δ32 variant, they achieved the cure of HIV infection in the ‘Berlin’, ‘London’, and ‘Düsseldorf’ patients, respectively. Also, studies performed in Spain by Enrich et al. (2018) and Germany, Poland, and the UK by Solloch et al. (2017) reinforced the importance of searching for CCR5Δ32 homozygous individuals in hematopoietic stem cell donor registries. Here, we conducted a similar investigation with Brazilian donors.

The CCR5Δ32 variant has a very heterogeneous distribution in different populations, even in the same country, as Brazil. According to Kulmann-Leal et al. (2021), the Brazilian CCR5Δ32 allele frequency is around 4-6%. In the present study, we found a CCR5Δ32 frequency of 7.4%, with ten individuals (0.72%) showing homozygous genotype, and these results answer our first research question. The increased CCR5Δ32 allele frequency observed in our samples may be due to the high density of European-descendent individuals in the south region of Brazil, as indicated by genetic and historical data (Callegari-Jacques et al., 2003; Lins et al., 2010). We also highlight that the CCR5Δ32 allele frequency observed in the present study is in agreement with our previous studies involving the CCR5Δ32 frequency (6-7%) in healthy sample groups from Rio Grande do Sul State (Vargas et al., 2006; Ellwanger et al., 2018; Ellwanger *et al.*, 2020c), highlighting the fact that this study encompassed a substantially larger sample size (more than a thousand individuals) than the previous works performed with other samples from Rio Grande do Sul (Vargas *et al.*, 2006; Ellwanger *et al.*, 2018; Ellwanger *et al.*, 2020c).

According to Solloch et al. (2017), the average Brazilian frequency of Δ32/Δ32 homozygous individuals is around 0.35%. It is important to highlight that Solloch *et al.* (2017) reported this frequency for only 570 individuals, and no information is given concerning the geographical or ethnic origin of this sample. Here, we found a higher frequency (0.72%). More than 5 million Brazilians are currently registered as bone marrow donors. Considering a minimum frequency of 0.35% (Solloch *et al.*, 2017) and a maximum frequency of 0.72% (this study), we would expect 17,500 to 36,000 of these voluntary donors homozygous for the CCR5Δ32 variant who could therefore participate in transplants for HIV-infected individuals with malignant hematological diseases. Nevertheless, when extrapolating these results to the Brazilian population in general, it should be noted that the frequencies of individuals carrying the CCR5Δ32 allele, in particular homozygous, vary along the country (Silva-Carvalho et al., 2016; Solloch *et al.*, 2017). Standardization of the genotyping of the CCR5Δ32 variant in all Brazilian stem cell donors, as well as considerations of populations composition would be required for a better estimate of these numbers in each region. Also, it is important to highlight that the estimate cited anteriorly only considers the genotypes of the CCR5Δ32 variant, and that a bone marrow transplant would only be viable with HLA system compatibility. Therefore, the real chance of finding a compatible donor who has the homozygous genotype for Δ32 is considerably lower. However, we stress that the intention of this work is to highlight the benefits of standardizing and including genotyping of the CCR5Δ32 variant in histocompatibility panel screenings, which we believe would be viable considering technical terms, with costs covered with resources from the Brazilian Unified Health System (*Sistema Único de Saúde* - SUS).

In this study, the *HLA-B* locus was the most polymorphic, with 30 alleles. The HLA-B locus is indeed very polymorphic, showing allele diversity superior to other HLA class I loci (Kiepiela et al., 2004). In general, in our study the evaluated *HLA-A*, *HLA-B*, and *HLA-DRB1* alleles and genotypes were homogeneously distributed between CCR5Δ32 carriers and non-carriers (*p*>0.05). The absence of an association of a given HLA allele/genotype with the CCR5Δ32 variant could be interpreted as advantageous since it would suggest that CCR5Δ32 donors are highly diversified in terms of their HLA genotypes, suggesting a wide range of possibilities concerning HLA/CCR5Δ32 matchings. 

Finally, we highlight that the sample size of this study, although considerably large, did not allow us to analyze the different *HLA* genotypes and alleles more comprehensively, representing a study limitation. However, as mentioned anteriorly, this is an exploratory study. Furthermore, due to the (expected) low frequency of the Δ32 allele, the number of CCR5Δ32 homozygotes was quite limited. Therefore, more studies with larger sample sizes are required to better understand potential interactions between *HLA* alleles and the CCR5Δ32 variant in the Brazilian population.

## Conclusion

The genotype and allele frequencies of the CCR5Δ32 in a sample of voluntary bone marrow donors of Rio Grande do Sul State (south Brazil) were reported in this study, as well as allele diversity of three *HLA* loci in the same sample. Considering the large number of individuals registered as bone marrow donors, here we show that it is possible to find a considerable amount of CCR5Δ32 homozygous donors who might donate bone marrow to HIV+ patients who need bone marrow transplantation due to a malignant hematological disease. We did not detect an association between *HLA*-specific genotypes and the CCR5Δ32 variant. 

## Supplementary material

The following online material is available for this article:

Table S1 -
*HLA-A* genotypes.

Table S2 - 
*HLA-B* genotypes.

Table S3 - 
*HLA-DRB1* genotypes.
