# Supplementary material for: CCR5Δ32 and HLA allele diversity in bone marrow donors from southern Brazil
Source: Genet Mol Biol. 2024 Jul 29;47(3):e20230198. doi: 10.1590/1678-4685-GMB-2023-0198 (PMC11285832; doi:10.1590/1678-4685-GMB-2023-0198)
Supplement: Table S1 - [file 1415-4757-GMB-47-03-e20230198-s1.pdf]

Genetics and Molecular Biology
https://doi.org/10.1590/1678-4685-GMB-2023-0198

Supplementary Material to "CCR5Δ32 and HLA allele diversity in bone marrow donors from southern Brazil"

Table S1 - HLA-A genotypes.

```
Genotype frequencies:

===============================
        Locus: HLA-A
===============================

Non-carriers:

Genotypes    Observed  Expected under HW equilibrium
     1 , 1      6         8.7474
     2 , 1      67       52.9498
     2 , 2      75       79.5647
     3 , 1      22       18.9838
     3 , 2      68       57.1521
     3 , 3      6        10.1950
    11 , 1      8         9.8641
    11 , 2      40       29.6967
    11 , 3      6        10.6470
    11 , 11     2         2.7400
    23 , 1      8         8.0030
    23 , 2      11       24.0935
    23 , 3      11        8.6381
    23 , 11     3         4.4884
    23 , 23     4         1.7996
    24 , 1      25       21.4032
    24 , 2      62       64.4362
    24 , 3      21       23.1019
    24 , 11     7        12.0039
    24 , 23     10        9.7390
    24 , 24     16       12.9665
    25 , 1      0         2.4195
    25 , 2      6         7.2841
    25 , 3      1         2.6115
    25 , 11     3         1.3570
    25 , 23     1         1.1009
    25 , 24     3         2.9444
    25 , 25     0         0.1600
    26 , 1      4         5.7696
    26 , 2      16       17.3698
    26 , 3      11        6.2275
    26 , 11     2         3.2358
    26 , 23     6         2.6253
    26 , 24     6         7.0212
    26 , 25     0         0.7937
    26 , 26     1         0.9311
    29 , 1      8        10.6086
    29 , 2      32       31.9380
```

29 , 3	9	11.4505
29 , 11	7	5.9498
29 , 23	5	4.8272
29 , 24	14	12.9099
29 , 25	4	1.4594
29 , 26	6	3.4801
29 , 29	2	3.1713
30 , 1	8	6.0487
30 , 2	17	18.2102
30 , 3	7	6.5288
30 , 11	3	3.3924
30 , 23	3	2.7523
30 , 24	5	7.3609
30 , 25	0	0.8321
30 , 26	1	1.9842
30 , 29	2	3.6484
30 , 30	1	1.0241
31 , 1	4	8.1891
31 , 2	27	24.6539
31 , 3	9	8.8390
31 , 11	2	4.5928
31 , 23	3	3.7262
31 , 24	12	9.9655
31 , 25	2	1.1265
31 , 26	3	2.6864
31 , 29	4	4.9394
31 , 30	2	2.8163
31 , 31	3	1.8848
32 , 1	4	7.2585
32 , 2	22	21.8523
32 , 3	8	7.8346
32 , 11	5	4.0709
32 , 23	5	3.3028
32 , 24	9	8.8331
32 , 25	1	0.9985
32 , 26	1	2.3811
32 , 29	4	4.3781
32 , 30	4	2.4963
32 , 31	1	3.3796
32 , 32	3	1.4786
33 , 1	8	6.0487
33 , 2	17	18.2102
33 , 3	6	6.5288
33 , 11	4	3.3924
33 , 23	1	2.7523
33 , 24	7	7.3609
33 , 25	2	0.8321
33 , 26	1	1.9842
33 , 29	4	3.6484
33 , 30	3	2.0803
33 , 31	4	2.8163
33 , 32	2	2.4963
33 , 33	1	1.0241
34 , 1	0	0.7445
34 , 2	2	2.2413
34 , 3	0	0.8035
34 , 11	0	0.4175
34 , 23	3	0.3387

34 , 24	2	0.9060
34 , 25	1	0.1024
34 , 26	0	0.2442
34 , 29	0	0.4490
34 , 30	0	0.2560
34 , 31	0	0.3466
34 , 32	0	0.3072
34 , 33	0	0.2560
34 , 34	0	0.0138
36 , 1	0	0.5583
36 , 2	2	1.6809
36 , 3	0	0.6027
36 , 11	0	0.3131
36 , 23	1	0.2541
36 , 24	1	0.6795
36 , 25	0	0.0768
36 , 26	0	0.1832
36 , 29	0	0.3368
36 , 30	1	0.1920
36 , 31	0	0.2600
36 , 32	0	0.2304
36 , 33	0	0.1920
36 , 34	0	0.0236
36 , 36	0	0.0074
66 , 1	2	1.3028
66 , 2	0	3.9222
66 , 3	2	1.4062
66 , 11	1	0.7307
66 , 23	1	0.5928
66 , 24	2	1.5854
66 , 25	1	0.1792
66 , 26	1	0.4274
66 , 29	2	0.7858
66 , 30	0	0.4481
66 , 31	0	0.6066
66 , 32	1	0.5377
66 , 33	0	0.4481
66 , 34	0	0.0551
66 , 36	1	0.0414
66 , 66	0	0.0448
68 , 1	9	9.9572
68 , 2	24	29.9769
68 , 3	10	10.7474
68 , 11	11	5.5844
68 , 23	5	4.5308
68 , 24	11	12.1172
68 , 25	1	1.3698
68 , 26	2	3.2664
68 , 29	9	6.0059
68 , 30	5	3.4244
68 , 31	8	4.6361
68 , 32	5	4.1093
68 , 33	3	3.4244
68 , 34	0	0.4215
68 , 36	0	0.3161
68 , 66	0	0.7376
68 , 68	1	2.7922
69 , 1	0	0.1861

69 , 2	1	0.5603
69 , 3	0	0.2009
69 , 11	0	0.1044
69 , 23	1	0.0847
69 , 24	0	0.2265
69 , 25	0	0.0256
69 , 26	0	0.0611
69 , 29	0	0.1123
69 , 30	0	0.0640
69 , 31	0	0.0867
69 , 32	0	0.0768
69 , 33	0	0.0640
69 , 34	0	0.0079
69 , 36	0	0.0059
69 , 66	0	0.0138
69 , 68	0	0.1054
69 , 69	0	0.0005
74 , 1	0	1.2097
74 , 2	5	3.6420
74 , 3	1	1.3058
74 , 11	0	0.6785
74 , 23	0	0.5505
74 , 24	1	1.4722
74 , 25	0	0.1664
74 , 26	0	0.3968
74 , 29	0	0.7297
74 , 30	2	0.4161
74 , 31	1	0.5633
74 , 32	0	0.4993
74 , 33	1	0.4161
74 , 34	0	0.0512
74 , 36	0	0.0384
74 , 66	0	0.0896
74 , 68	2	0.6849
74 , 69	0	0.0128
74 , 74	0	0.0384

Carriers:

Genotypes	Observed	Expected under HW equilibrium
1 , 1	2	2.2478
2 , 1	10	11.8732
2 , 2	16	15.1383
3 , 1	5	3.1124
3 , 2	8	8.0144
3 , 3	3	1.0115
11 , 1	0	1.8444
11 , 2	5	4.7493
11 , 3	1	1.2450
11 , 11	1	0.3458
23 , 1	2	1.2680
23 , 2	2	3.2651
23 , 3	2	0.8559
23 , 11	2	0.5072
23 , 23	0	0.1585
24 , 1	5	3.9193
24 , 2	15	10.0922

24 , 3	1	2.6455
24 , 11	1	1.5677
24 , 23	2	1.0778
24 , 24	0	1.6167
25 , 1	1	0.8069
25 , 2	2	2.0778
25 , 3	0	0.5447
25 , 11	0	0.3228
25 , 23	0	0.2219
25 , 24	1	0.6859
25 , 25	0	0.0605
26 , 1	0	1.1527
26 , 2	1	2.9683
26 , 3	1	0.7781
26 , 11	0	0.4611
26 , 23	0	0.3170
26 , 24	1	0.9798
26 , 25	1	0.2017
26 , 26	0	0.1297
29 , 1	0	2.0749
29 , 2	6	5.3429
29 , 3	2	1.4006
29 , 11	0	0.8300
29 , 23	0	0.5706
29 , 24	1	1.7637
29 , 25	1	0.3631
29 , 26	0	0.5187
29 , 29	1	0.4409
30 , 1	1	1.1527
30 , 2	2	2.9683
30 , 3	0	0.7781
30 , 11	0	0.4611
30 , 23	1	0.3170
30 , 24	1	0.9798
30 , 25	0	0.2017
30 , 26	2	0.2882
30 , 29	0	0.5187
30 , 30	0	0.1297
31 , 1	2	1.1527
31 , 2	3	2.9683
31 , 3	0	0.7781
31 , 11	1	0.4611
31 , 23	0	0.3170
31 , 24	0	0.9798
31 , 25	0	0.2017
31 , 26	1	0.2882
31 , 29	1	0.5187
31 , 30	0	0.2882
31 , 31	0	0.1297
32 , 1	4	2.6513
32 , 2	6	6.8271
32 , 3	0	1.7896
32 , 11	0	1.0605
32 , 23	0	0.7291
32 , 24	2	2.2536
32 , 25	1	0.4640
32 , 26	0	0.6628
32 , 29	2	1.1931

32 , 30	3	0.6628
32 , 31	0	0.6628
32 , 32	0	0.7291
33 , 1	1	1.4986
33 , 2	3	3.8588
33 , 3	0	1.0115
33 , 11	2	0.5994
33 , 23	0	0.4121
33 , 24	2	1.2738
33 , 25	0	0.2622
33 , 26	2	0.3746
33 , 29	0	0.6744
33 , 30	0	0.3746
33 , 31	2	0.3746
33 , 32	1	0.8617
33 , 33	0	0.2248
36 , 1	0	0.1153
36 , 2	1	0.2968
36 , 3	0	0.0778
36 , 11	0	0.0461
36 , 23	0	0.0317
36 , 24	0	0.0980
36 , 25	0	0.0202
36 , 26	0	0.0288
36 , 29	0	0.0519
36 , 30	0	0.0288
36 , 31	0	0.0288
36 , 32	0	0.0663
36 , 33	0	0.0375
36 , 36	0	0.0000
66 , 1	0	0.2305
66 , 2	0	0.5937
66 , 3	0	0.1556
66 , 11	0	0.0922
66 , 23	0	0.0634
66 , 24	0	0.1960
66 , 25	0	0.0403
66 , 26	1	0.0576
66 , 29	1	0.1037
66 , 30	0	0.0576
66 , 31	0	0.0576
66 , 32	0	0.1326
66 , 33	0	0.0749
66 , 36	0	0.0058
66 , 66	0	0.0029
68 , 1	5	2.3055
68 , 2	5	5.9366
68 , 3	1	1.5562
68 , 11	2	0.9222
68 , 23	0	0.6340
68 , 24	2	1.9597
68 , 25	0	0.4035
68 , 26	0	0.5764
68 , 29	2	1.0375
68 , 30	0	0.5764
68 , 31	0	0.5764
68 , 32	3	1.3256
68 , 33	0	0.7493

68 , 36	0	0.0576
68 , 66	0	0.1153
68 , 68	0	0.5476
74 , 1	0	0.3458
74 , 2	2	0.8905
74 , 3	0	0.2334
74 , 11	0	0.1383
74 , 23	0	0.0951
74 , 24	0	0.2939
74 , 25	0	0.0605
74 , 26	0	0.0865
74 , 29	0	0.1556
74 , 30	0	0.0865
74 , 31	0	0.0865
74 , 32	1	0.1988
74 , 33	0	0.1124
74 , 36	0	0.0086
74 , 66	0	0.0173
74 , 68	0	0.1729
74 , 74	0	0.0086
