# Supplementary material for: CCR5Δ32 and HLA allele diversity in bone marrow donors from southern Brazil
Source: Genet Mol Biol. 2024 Jul 29;47(3):e20230198. doi: 10.1590/1678-4685-GMB-2023-0198 (PMC11285832; doi:10.1590/1678-4685-GMB-2023-0198)
Supplement: Table S2 - [file 1415-4757-GMB-47-03-e20230198-s2.pdf]

Genetics and Molecular Biology
https://doi.org/10.1590/1678-4685-GMB-2023-0198

Supplementary Material to "CCR5Δ32 and HLA allele diversity in bone marrow donors from southern Brazil"

Table S2- HLA-B genotypes.

Genotype frequencies:

```
================================
        Locus: HLA-B
================================
```

Non-carriers:

Genotypes	Observed	Expected under HW equilibrium
7 , 7	12	7.4963
8 , 7	0	9.8227
8 , 8	2	3.1713
13 , 7	7	4.6529
13 , 8	1	3.0310
13 , 13	0	0.7046
14 , 7	8	9.5643
14 , 8	8	6.2304
14 , 13	5	2.9513
14 , 14	3	3.0059
15 , 7	11	14.3033
15 , 8	15	9.3176
15 , 13	5	4.4136
15 , 14	7	9.0724
15 , 15	4	6.7430
18 , 7	8	9.2196
18 , 8	11	6.0059
18 , 13	2	2.8449
18 , 14	5	5.8479
18 , 15	8	8.7454
18 , 18	5	2.7922
27 , 7	3	4.4806
27 , 8	4	2.9188
27 , 13	3	1.3826
27 , 14	1	2.8419
27 , 15	3	4.2501
27 , 18	2	2.7395
27 , 27	1	0.6529
35 , 7	23	19.3870
35 , 8	10	12.6292
35 , 13	3	5.9823
35 , 14	11	12.2969
35 , 15	18	18.3900
35 , 18	12	11.8538
35 , 27	6	5.7607
35 , 35	10	12.4077
37 , 7	4	1.7233

37 , 8		1	1.1226
37 , 13		0	0.5318
37 , 14		1	1.0931
37 , 15		1	1.6347
37 , 18		0	1.0537
37 , 27		0	0.5121
37 , 35		3	2.2157
37 , 37		0	0.0935
38 , 7		2	4.9114
38 , 8		5	3.1994
38 , 13		2	1.5155
38 , 14		6	3.1152
38 , 15		7	4.6588
38 , 18		3	3.0030
38 , 27		1	1.4594
38 , 35		4	6.3146
38 , 37		0	0.5613
38 , 38		1	0.7858
39 , 7		1	2.9296
39 , 8		5	1.9084
39 , 13		1	0.9040
39 , 14		3	1.8582
39 , 15		1	2.7789
39 , 18		0	1.7912
39 , 27		2	0.8705
39 , 35		4	3.7666
39 , 37		1	0.3348
39 , 38		2	0.9542
39 , 39		0	0.2762
40 , 7		8	10.2536
40 , 8		9	6.6795
40 , 13		3	3.1640
40 , 14		9	6.5037
40 , 15		7	9.7262
40 , 18		8	6.2693
40 , 27		7	3.0468
40 , 35		13	13.1832
40 , 37		0	1.1718
40 , 38		3	3.3397
40 , 39		0	1.9921
40 , 40		4	3.4569
41 , 7		2	1.8095
41 , 8		1	1.1787
41 , 13		0	0.5583
41 , 14		2	1.1477
41 , 15		1	1.7164
41 , 18		2	1.1064
41 , 27		1	0.5377
41 , 35		2	2.3264
41 , 37		0	0.2068
41 , 38		0	0.5894
41 , 39		2	0.3516
41 , 40		1	1.2304
41 , 41		0	0.1034
42 , 7		1	1.1201
42 , 8		0	0.7297
42 , 13		1	0.3456
42 , 14		1	0.7105

42	, 15	2	1.0625
42	, 18	1	0.6849
42	, 27	1	0.3328
42	, 35	1	1.4402
42	, 37	0	0.1280
42	, 38	1	0.3648
42	, 39	0	0.2176
42	, 40	1	0.7617
42	, 41	0	0.1344
42	, 42	0	0.0384
44	, 7	31	23.1782
44	, 8	23	15.0990
44	, 13	5	7.1521
44	, 14	9	14.7016
44	, 15	26	21.9862
44	, 18	10	14.1718
44	, 27	6	6.8872
44	, 35	40	29.8006
44	, 37	4	2.6489
44	, 38	4	7.5495
44	, 39	2	4.5032
44	, 40	13	15.7612
44	, 41	3	2.7814
44	, 42	0	1.7218
44	, 44	13	17.7479
45	, 7	3	2.4126
45	, 8	3	1.5716
45	, 13	0	0.7445
45	, 14	4	1.5303
45	, 15	3	2.2885
45	, 18	2	1.4751
45	, 27	0	0.7169
45	, 35	1	3.1019
45	, 37	0	0.2757
45	, 38	0	0.7858
45	, 39	1	0.4687
45	, 40	3	1.6406
45	, 41	0	0.2895
45	, 42	0	0.1792
45	, 44	4	3.7085
45	, 45	0	0.1861
46	, 7	0	0.0862
46	, 8	1	0.0561
46	, 13	0	0.0266
46	, 14	0	0.0547
46	, 15	0	0.0817
46	, 18	0	0.0527
46	, 27	0	0.0256
46	, 35	0	0.1108
46	, 37	0	0.0098
46	, 38	0	0.0281
46	, 39	0	0.0167
46	, 40	0	0.0586
46	, 41	0	0.0103
46	, 42	0	0.0064
46	, 44	0	0.1324
46	, 45	0	0.0138
46	, 46	0	0.0000

47 , 7	0	0.7755
47 , 8	1	0.5052
47 , 13	0	0.2393
47 , 14	0	0.4919
47 , 15	0	0.7356
47 , 18	1	0.4742
47 , 27	1	0.2304
47 , 35	2	0.9970
47 , 37	1	0.0886
47 , 38	1	0.2526
47 , 39	0	0.1507
47 , 40	1	0.5273
47 , 41	0	0.0931
47 , 42	0	0.0576
47 , 44	1	1.1920
47 , 45	0	0.1241
47 , 46	0	0.0044
47 , 47	0	0.0177
48 , 7	1	2.1541
48 , 8	3	1.4032
48 , 13	0	0.6647
48 , 14	3	1.3663
48 , 15	0	2.0433
48 , 18	0	1.3171
48 , 27	0	0.6401
48 , 35	5	2.7696
48 , 37	0	0.2462
48 , 38	0	0.7016
48 , 39	0	0.4185
48 , 40	2	1.4648
48 , 41	0	0.2585
48 , 42	0	0.1600
48 , 44	6	3.3112
48 , 45	0	0.3447
48 , 46	0	0.0123
48 , 47	0	0.1108
48 , 48	0	0.1477
49 , 7	3	4.4806
49 , 8	1	2.9188
49 , 13	4	1.3826
49 , 14	2	2.8419
49 , 15	4	4.2501
49 , 18	2	2.7395
49 , 27	0	1.3314
49 , 35	9	5.7607
49 , 37	0	0.5121
49 , 38	2	1.4594
49 , 39	2	0.8705
49 , 40	0	3.0468
49 , 41	0	0.5377
49 , 42	0	0.3328
49 , 44	9	6.8872
49 , 45	0	0.7169
49 , 46	0	0.0256
49 , 47	0	0.2304
49 , 48	1	0.6401
49 , 49	1	0.6529
50 , 7	4	3.1881

50 , 8	4	2.0768
50 , 13	1	0.9838
50 , 14	1	2.0222
50 , 15	3	3.0241
50 , 18	1	1.9493
50 , 27	1	0.9473
50 , 35	4	4.0990
50 , 37	0	0.3644
50 , 38	2	1.0384
50 , 39	1	0.6194
50 , 40	4	2.1679
50 , 41	0	0.3826
50 , 42	0	0.2368
50 , 44	5	4.9005
50 , 45	0	0.5101
50 , 46	0	0.0182
50 , 47	0	0.1640
50 , 48	2	0.4554
50 , 49	0	0.9473
50 , 50	0	0.3279
51 , 7	14	14.2171
51 , 8	0	9.2614
51 , 13	8	4.3870
51 , 14	9	9.0177
51 , 15	17	13.4860
51 , 18	11	8.6928
51 , 27	4	4.2245
51 , 35	17	18.2792
51 , 37	2	1.6248
51 , 38	7	4.6307
51 , 39	4	2.7622
51 , 40	10	9.6677
51 , 41	2	1.7061
51 , 42	2	1.0561
51 , 44	18	21.8538
51 , 45	0	2.2747
51 , 46	0	0.0812
51 , 47	0	0.7312
51 , 48	2	2.0310
51 , 49	3	4.2245
51 , 50	3	3.0059
51 , 51	6	6.6617
52 , 7	2	2.2403
52 , 8	0	1.4594
52 , 13	1	0.6913
52 , 14	1	1.4210
52 , 15	4	2.1251
52 , 18	0	1.3698
52 , 27	1	0.6657
52 , 35	3	2.8804
52 , 37	0	0.2560
52 , 38	1	0.7297
52 , 39	0	0.4353
52 , 40	3	1.5234
52 , 41	0	0.2688
52 , 42	0	0.1664
52 , 44	3	3.4436
52 , 45	1	0.3584

52 ,	46	0	0.0128
52 ,	47	0	0.1152
52 ,	48	0	0.3200
52 ,	49	2	0.6657
52 ,	50	0	0.4737
52 ,	51	2	2.1123
52 ,	52	0	0.1600
53 ,	7	3	1.9818
53 ,	8	0	1.2910
53 ,	13	0	0.6115
53 ,	14	1	1.2570
53 ,	15	3	1.8799
53 ,	18	0	1.2117
53 ,	27	0	0.5889
53 ,	35	5	2.5480
53 ,	37	0	0.2265
53 ,	38	0	0.6455
53 ,	39	1	0.3850
53 ,	40	0	1.3476
53 ,	41	0	0.2378
53 ,	42	0	0.1472
53 ,	44	5	3.0463
53 ,	45	1	0.3171
53 ,	46	0	0.0113
53 ,	47	0	0.1019
53 ,	48	0	0.2831
53 ,	49	0	0.5889
53 ,	50	0	0.4190
53 ,	51	0	1.8685
53 ,	52	0	0.2944
53 ,	53	0	0.1246
55 ,	7	1	1.9818
55 ,	8	0	1.2910
55 ,	13	1	0.6115
55 ,	14	1	1.2570
55 ,	15	1	1.8799
55 ,	18	1	1.2117
55 ,	27	1	0.5889
55 ,	35	2	2.5480
55 ,	37	1	0.2265
55 ,	38	0	0.6455
55 ,	39	1	0.3850
55 ,	40	0	1.3476
55 ,	41	0	0.2378
55 ,	42	0	0.1472
55 ,	44	7	3.0463
55 ,	45	0	0.3171
55 ,	46	0	0.0113
55 ,	47	0	0.1019
55 ,	48	0	0.2831
55 ,	49	0	0.5889
55 ,	50	0	0.4190
55 ,	51	3	1.8685
55 ,	52	0	0.2944
55 ,	53	1	0.2605
55 ,	55	1	0.1246
56 ,	7	3	0.9478
56 ,	8	0	0.6174

56 , 13	0	0.2925
56 , 14	1	0.6012
56 , 15	2	0.8991
56 , 18	0	0.5795
56 , 27	0	0.2816
56 , 35	0	1.2186
56 , 37	0	0.1083
56 , 38	0	0.3087
56 , 39	0	0.1841
56 , 40	2	0.6445
56 , 41	0	0.1137
56 , 42	0	0.0704
56 , 44	0	1.4569
56 , 45	0	0.1516
56 , 46	0	0.0054
56 , 47	0	0.0487
56 , 48	0	0.1354
56 , 49	0	0.2816
56 , 50	0	0.2004
56 , 51	1	0.8936
56 , 52	0	0.1408
56 , 53	0	0.1246
56 , 55	0	0.1246
56 , 56	0	0.0271
57 , 7	5	4.3944
57 , 8	4	2.8626
57 , 13	1	1.3560
57 , 14	3	2.7873
57 , 15	2	4.1684
57 , 18	4	2.6869
57 , 27	0	1.3058
57 , 35	4	5.6499
57 , 37	0	0.5022
57 , 38	2	1.4313
57 , 39	0	0.8538
57 , 40	4	2.9882
57 , 41	1	0.5273
57 , 42	0	0.3264
57 , 44	4	6.7548
57 , 45	0	0.7031
57 , 46	0	0.0251
57 , 47	0	0.2260
57 , 48	0	0.6278
57 , 49	3	1.3058
57 , 50	1	0.9291
57 , 51	8	4.1433
57 , 52	2	0.6529
57 , 53	1	0.5775
57 , 55	0	0.5775
57 , 56	1	0.2762
57 , 57	0	0.6278
58 , 7	2	3.3604
58 , 8	0	2.1891
58 , 13	0	1.0369
58 , 14	2	2.1315
58 , 15	7	3.1876
58 , 18	3	2.0547
58 , 27	2	0.9985

58 , 35	2	4.3205
58 , 37	1	0.3840
58 , 38	0	1.0945
58 , 39	0	0.6529
58 , 40	0	2.2851
58 , 41	1	0.4032
58 , 42	1	0.2496
58 , 44	4	5.1654
58 , 45	2	0.5377
58 , 46	0	0.0192
58 , 47	0	0.1728
58 , 48	0	0.4801
58 , 49	2	0.9985
58 , 50	0	0.7105
58 , 51	6	3.1684
58 , 52	0	0.4993
58 , 53	2	0.4417
58 , 55	0	0.4417
58 , 56	1	0.2112
58 , 57	1	0.9793
58 , 58	0	0.3648
73 , 7	1	0.0862
73 , 8	0	0.0561
73 , 13	0	0.0266
73 , 14	0	0.0547
73 , 15	0	0.0817
73 , 18	0	0.0527
73 , 27	0	0.0256
73 , 35	0	0.1108
73 , 37	0	0.0098
73 , 38	0	0.0281
73 , 39	0	0.0167
73 , 40	0	0.0586
73 , 41	0	0.0103
73 , 42	0	0.0064
73 , 44	0	0.1324
73 , 45	0	0.0138
73 , 46	0	0.0005
73 , 47	0	0.0044
73 , 48	0	0.0123
73 , 49	0	0.0256
73 , 50	0	0.0182
73 , 51	0	0.0812
73 , 52	0	0.0128
73 , 53	0	0.0113
73 , 55	0	0.0113
73 , 56	0	0.0054
73 , 57	0	0.0251
73 , 58	0	0.0192
73 , 73	0	0.0000
81 , 7	0	0.3447
81 , 8	0	0.2245
81 , 13	0	0.1064
81 , 14	1	0.2186
81 , 15	0	0.3269
81 , 18	0	0.2107
81 , 27	0	0.1024
81 , 35	1	0.4431

81 , 37	0	0.0394
81 , 38	0	0.1123
81 , 39	0	0.0670
81 , 40	0	0.2344
81 , 41	0	0.0414
81 , 42	0	0.0256
81 , 44	1	0.5298
81 , 45	0	0.0551
81 , 46	0	0.0020
81 , 47	0	0.0177
81 , 48	0	0.0492
81 , 49	1	0.1024
81 , 50	0	0.0729
81 , 51	0	0.3250
81 , 52	0	0.0512
81 , 53	0	0.0453
81 , 55	0	0.0453
81 , 56	0	0.0217
81 , 57	0	0.1004
81 , 58	0	0.0768
81 , 73	0	0.0020
81 , 81	0	0.0030

Carriers:

Genotypes	Observed	Expected under HW equilibrium
7 , 7	1	1.1700
8 , 7	0	1.5879
8 , 8	1	0.4928
13 , 7	3	0.9193
13 , 8	0	0.6023
13 , 13	0	0.1585
14 , 7	1	1.4207
14 , 8	0	0.9308
14 , 13	0	0.5389
14 , 14	0	0.3919
15 , 7	4	3.3429
15 , 8	1	2.1902
15 , 13	3	1.2680
15 , 14	3	1.9597
15 , 15	3	2.2478
18 , 7	0	1.5879
18 , 8	2	1.0403
18 , 13	1	0.6023
18 , 14	1	0.9308
18 , 15	3	2.1902
18 , 18	0	0.4928
27 , 7	2	1.1700
27 , 8	0	0.7666
27 , 13	0	0.4438
27 , 14	3	0.6859
27 , 15	0	1.6138
27 , 18	0	0.7666
27 , 27	0	0.2622
35 , 7	6	3.2594
35 , 8	6	2.1354
35 , 13	1	1.2363

35 ,	14	1	1.9107
35 ,	15	4	4.4957
35 ,	18	1	2.1354
35 ,	27	2	1.5735
35 ,	35	1	2.1354
37 ,	7	0	0.3343
37 ,	8	0	0.2190
37 ,	13	0	0.1268
37 ,	14	0	0.1960
37 ,	15	0	0.4611
37 ,	18	0	0.2190
37 ,	27	1	0.1614
37 ,	35	0	0.4496
37 ,	37	0	0.0173
38 ,	7	0	1.0865
38 ,	8	0	0.7118
38 ,	13	0	0.4121
38 ,	14	2	0.6369
38 ,	15	0	1.4986
38 ,	18	2	0.7118
38 ,	27	1	0.5245
38 ,	35	0	1.4611
38 ,	37	0	0.1499
38 ,	38	1	0.2248
39 ,	7	0	0.7522
39 ,	8	1	0.4928
39 ,	13	0	0.2853
39 ,	14	1	0.4409
39 ,	15	0	1.0375
39 ,	18	2	0.4928
39 ,	27	1	0.3631
39 ,	35	1	1.0115
39 ,	37	0	0.1037
39 ,	38	0	0.3372
39 ,	39	0	0.1037
40 ,	7	1	0.9193
40 ,	8	1	0.6023
40 ,	13	0	0.3487
40 ,	14	0	0.5389
40 ,	15	0	1.2680
40 ,	18	1	0.6023
40 ,	27	0	0.4438
40 ,	35	2	1.2363
40 ,	37	0	0.1268
40 ,	38	1	0.4121
40 ,	39	1	0.2853
40 ,	40	0	0.1585
41 ,	7	0	0.5014
41 ,	8	0	0.3285
41 ,	13	0	0.1902
41 ,	14	0	0.2939
41 ,	15	1	0.6916
41 ,	18	1	0.3285
41 ,	27	0	0.2421
41 ,	35	2	0.6744
41 ,	37	0	0.0692
41 ,	38	0	0.2248
41 ,	39	0	0.1556

41 , 40		0	0.1902
41 , 41		0	0.0432
42 , 7		0	0.1671
42 , 8		0	0.1095
42 , 13		0	0.0634
42 , 14		0	0.0980
42 , 15		0	0.2305
42 , 18		0	0.1095
42 , 27		0	0.0807
42 , 35		0	0.2248
42 , 37		0	0.0231
42 , 38		0	0.0749
42 , 39		0	0.0519
42 , 40		0	0.0634
42 , 41		0	0.0346
42 , 42		0	0.0029
44 , 7		2	2.2565
44 , 8		1	1.4784
44 , 13		2	0.8559
44 , 14		2	1.3228
44 , 15		1	3.1124
44 , 18		1	1.4784
44 , 27		1	1.0893
44 , 35		3	3.0346
44 , 37		2	0.3112
44 , 38		1	1.0115
44 , 39		1	0.7003
44 , 40		0	0.8559
44 , 41		1	0.4669
44 , 42		1	0.1556
44 , 44		1	1.0115
45 , 7		0	0.4179
45 , 8		0	0.2738
45 , 13		0	0.1585
45 , 14		1	0.2450
45 , 15		1	0.5764
45 , 18		1	0.2738
45 , 27		0	0.2017
45 , 35		2	0.5620
45 , 37		0	0.0576
45 , 38		0	0.1873
45 , 39		0	0.1297
45 , 40		0	0.1585
45 , 41		0	0.0865
45 , 42		0	0.0288
45 , 44		0	0.3890
45 , 45		0	0.0288
47 , 7		0	0.0836
47 , 8		0	0.0548
47 , 13		0	0.0317
47 , 14		0	0.0490
47 , 15		1	0.1153
47 , 18		0	0.0548
47 , 27		0	0.0403
47 , 35		0	0.1124
47 , 37		0	0.0115
47 , 38		0	0.0375
47 , 39		0	0.0259

47 , 40	0	0.0317
47 , 41	0	0.0173
47 , 42	0	0.0058
47 , 44	0	0.0778
47 , 45	0	0.0144
47 , 47	0	0.0000
48 , 7	0	0.1671
48 , 8	0	0.1095
48 , 13	0	0.0634
48 , 14	0	0.0980
48 , 15	0	0.2305
48 , 18	0	0.1095
48 , 27	0	0.0807
48 , 35	0	0.2248
48 , 37	0	0.0231
48 , 38	0	0.0749
48 , 39	0	0.0519
48 , 40	0	0.0634
48 , 41	1	0.0346
48 , 42	0	0.0115
48 , 44	0	0.1556
48 , 45	0	0.0288
48 , 47	0	0.0058
48 , 48	0	0.0029
49 , 7	0	0.7522
49 , 8	1	0.4928
49 , 13	1	0.2853
49 , 14	1	0.4409
49 , 15	0	1.0375
49 , 18	1	0.4928
49 , 27	1	0.3631
49 , 35	0	1.0115
49 , 37	0	0.1037
49 , 38	0	0.3372
49 , 39	0	0.2334
49 , 40	0	0.2853
49 , 41	0	0.1556
49 , 42	0	0.0519
49 , 44	0	0.7003
49 , 45	0	0.1297
49 , 47	0	0.0259
49 , 48	0	0.0519
49 , 49	0	0.1037
50 , 7	0	1.0029
50 , 8	1	0.6571
50 , 13	0	0.3804
50 , 14	0	0.5879
50 , 15	4	1.3833
50 , 18	0	0.6571
50 , 27	1	0.4841
50 , 35	1	1.3487
50 , 37	0	0.1383
50 , 38	1	0.4496
50 , 39	1	0.3112
50 , 40	0	0.3804
50 , 41	0	0.2075
50 , 42	0	0.0692
50 , 44	1	0.9337

50 , 45	0	0.1729
50 , 47	0	0.0346
50 , 48	1	0.0692
50 , 49	0	0.3112
50 , 50	0	0.1902
51 , 7	4	2.7579
51 , 8	0	1.8069
51 , 13	0	1.0461
51 , 14	1	1.6167
51 , 15	6	3.8040
51 , 18	1	1.8069
51 , 27	1	1.3314
51 , 35	2	3.7089
51 , 37	1	0.3804
51 , 38	1	1.2363
51 , 39	0	0.8559
51 , 40	2	1.0461
51 , 41	0	0.5706
51 , 42	0	0.1902
51 , 44	3	2.5677
51 , 45	0	0.4755
51 , 47	0	0.0951
51 , 48	0	0.1902
51 , 49	2	0.8559
51 , 50	1	1.1412
51 , 51	2	1.5216
52 , 7	0	0.2507
52 , 8	0	0.1643
52 , 13	0	0.0951
52 , 14	0	0.1470
52 , 15	1	0.3458
52 , 18	0	0.1643
52 , 27	0	0.1210
52 , 35	0	0.3372
52 , 37	0	0.0346
52 , 38	1	0.1124
52 , 39	0	0.0778
52 , 40	0	0.0951
52 , 41	0	0.0519
52 , 42	1	0.0173
52 , 44	0	0.2334
52 , 45	0	0.0432
52 , 47	0	0.0086
52 , 48	0	0.0173
52 , 49	0	0.0778
52 , 50	0	0.1037
52 , 51	0	0.2853
52 , 52	0	0.0086
53 , 7	2	0.4179
53 , 8	0	0.2738
53 , 13	0	0.1585
53 , 14	0	0.2450
53 , 15	1	0.5764
53 , 18	0	0.2738
53 , 27	0	0.2017
53 , 35	1	0.5620
53 , 37	0	0.0576
53 , 38	0	0.1873

53 , 39	0	0.1297
53 , 40	1	0.1585
53 , 41	0	0.0865
53 , 42	0	0.0288
53 , 44	0	0.3890
53 , 45	0	0.0720
53 , 47	0	0.0144
53 , 48	0	0.0288
53 , 49	0	0.1297
53 , 50	0	0.1729
53 , 51	0	0.4755
53 , 52	0	0.0432
53 , 53	0	0.0288
55 , 7	0	0.3343
55 , 8	0	0.2190
55 , 13	0	0.1268
55 , 14	0	0.1960
55 , 15	0	0.4611
55 , 18	0	0.2190
55 , 27	0	0.1614
55 , 35	0	0.4496
55 , 37	0	0.0461
55 , 38	1	0.1499
55 , 39	0	0.1037
55 , 40	0	0.1268
55 , 41	0	0.0692
55 , 42	0	0.0231
55 , 44	1	0.3112
55 , 45	0	0.0576
55 , 47	0	0.0115
55 , 48	0	0.0231
55 , 49	1	0.1037
55 , 50	0	0.1383
55 , 51	1	0.3804
55 , 52	0	0.0346
55 , 53	0	0.0576
55 , 55	0	0.0173
56 , 7	1	0.0836
56 , 8	0	0.0548
56 , 13	0	0.0317
56 , 14	0	0.0490
56 , 15	0	0.1153
56 , 18	0	0.0548
56 , 27	0	0.0403
56 , 35	0	0.1124
56 , 37	0	0.0115
56 , 38	0	0.0375
56 , 39	0	0.0259
56 , 40	0	0.0317
56 , 41	0	0.0173
56 , 42	0	0.0058
56 , 44	0	0.0778
56 , 45	0	0.0144
56 , 47	0	0.0029
56 , 48	0	0.0058
56 , 49	0	0.0259
56 , 50	0	0.0346
56 , 51	0	0.0951

56 , 52	0	0.0086
56 , 53	0	0.0144
56 , 55	0	0.0115
56 , 56	0	0.0000
57 , 7	1	0.7522
57 , 8	2	0.4928
57 , 13	0	0.2853
57 , 14	0	0.4409
57 , 15	0	1.0375
57 , 18	1	0.4928
57 , 27	0	0.3631
57 , 35	1	1.0115
57 , 37	0	0.1037
57 , 38	0	0.3372
57 , 39	0	0.2334
57 , 40	1	0.2853
57 , 41	0	0.1556
57 , 42	0	0.0519
57 , 44	0	0.7003
57 , 45	0	0.1297
57 , 47	0	0.0259
57 , 48	0	0.0519
57 , 49	0	0.2334
57 , 50	0	0.3112
57 , 51	3	0.8559
57 , 52	0	0.0778
57 , 53	0	0.1297
57 , 55	0	0.1037
57 , 56	0	0.0259
57 , 57	0	0.1037
58 , 7	0	0.3343
58 , 8	1	0.2190
58 , 13	0	0.1268
58 , 14	0	0.1960
58 , 15	0	0.4611
58 , 18	0	0.2190
58 , 27	0	0.1614
58 , 35	1	0.4496
58 , 37	0	0.0461
58 , 38	0	0.1499
58 , 39	0	0.1037
58 , 40	0	0.1268
58 , 41	0	0.0692
58 , 42	0	0.0231
58 , 44	1	0.3112
58 , 45	0	0.0576
58 , 47	0	0.0115
58 , 48	0	0.0231
58 , 49	1	0.1037
58 , 50	0	0.1383
58 , 51	0	0.3804
58 , 52	0	0.0346
58 , 53	0	0.0576
58 , 55	0	0.0461
58 , 56	0	0.0115
58 , 57	0	0.1037
58 , 58	0	0.0173
