# Supplementary material for: CCR5Δ32 and HLA allele diversity in bone marrow donors from southern Brazil
Source: Genet Mol Biol. 2024 Jul 29;47(3):e20230198. doi: 10.1590/1678-4685-GMB-2023-0198 (PMC11285832; doi:10.1590/1678-4685-GMB-2023-0198)
Supplement: Table S3 - [file 1415-4757-GMB-47-03-e20230198-s3.pdf]

Genetics and Molecular Biology
https://doi.org/10.1590/1678-4685-GMB-2023-0198

Supplementary Material to "CCR5Δ32 and HLA allele diversity in bone marrow donors from southern Brazil"

Table S3 - HLA-DRB1 genotypes.

```
Genotype frequencies:

===============================
       Locus: HLA-DRB1
===============================

Non-carriers:

Genotypes    Observed  Expected under HW equilibrium
     1 , 1      8        9.9956
     3 , 1      15      18.8971
     3 , 3      12       8.8405
     4 , 1      33      23.8700
     4 , 3      19      22.4520
     4 , 4      12      14.1211
     7 , 1      24      29.6386
     7 , 3      40      27.8779
     7 , 4      36      35.2142
     7 , 7      16      21.7888
     8 , 1      13      10.6420
     8 , 3      7       10.0098
     8 , 4      13      12.6440
     8 , 7      16      15.6997
     8 , 8      3        2.7922
     9 , 1      5        4.3762
     9 , 3      6        4.1162
     9 , 4      4        5.1994
     9 , 7      8        6.4559
     9 , 8      1        2.3181
     9 , 9      0        0.4658
    10 , 1      4        2.6854
    10 , 3      2        2.5258
    10 , 4      3        3.1905
    10 , 7      5        3.9616
    10 , 8      4        1.4225
    10 , 9      1        0.5849
    10 , 10     0        0.1728
    11 , 1      23      26.4559
    11 , 3      22      24.8843
    11 , 4      28      31.4328
    11 , 7      35      39.0290
    11 , 8      17      14.0138
    11 , 9      5        5.7627
    11 , 10     0        3.5362
    11 , 11     25      17.3535
    12 , 1      2        2.6854
    12 , 3      2        2.5258
```

12 , 4	4	3.1905
12 , 7	9	3.9616
12 , 8	1	1.4225
12 , 9	0	0.5849
12 , 10	0	0.3589
12 , 11	4	3.5362
12 , 12	0	0.1728
13 , 1	28	25.6603
13 , 3	30	24.1359
13 , 4	27	30.4874
13 , 7	36	37.8552
13 , 8	9	13.5923
13 , 9	3	5.5894
13 , 10	1	3.4298
13 , 11	36	33.7903
13 , 12	1	3.4298
13 , 13	17	16.3235
14 , 1	9	9.8464
14 , 3	5	9.2614
14 , 4	17	11.6987
14 , 7	14	14.5258
14 , 8	7	5.2157
14 , 9	1	2.1448
14 , 10	2	1.3161
14 , 11	12	12.9660
14 , 12	2	1.3161
14 , 13	14	12.5761
14 , 14	3	2.3885
15 , 1	25	20.6873
15 , 3	10	19.4584
15 , 4	22	24.5790
15 , 7	33	30.5190
15 , 8	10	10.9581
15 , 9	7	4.5062
15 , 10	3	2.7651
15 , 11	27	27.2418
15 , 12	1	2.7651
15 , 13	32	26.4225
15 , 14	5	10.1388
15 , 15	15	10.5997
16 , 1	5	6.5643
16 , 3	8	6.1743
16 , 4	10	7.7991
16 , 7	10	9.6839
16 , 8	3	3.4771
16 , 9	3	1.4298
16 , 10	2	0.8774
16 , 11	7	8.6440
16 , 12	1	0.8774
16 , 13	7	8.3840
16 , 14	5	3.2171
16 , 15	3	6.7592
16 , 16	1	1.0561

____

Carriers:

Genotypes   Observed   Expected under HW equilibrium

1 , 1	2	2.1354
3 , 1	1	3.7089
3 , 3	2	1.5216
4 , 1	5	4.7205
4 , 3	5	3.9942
4 , 4	2	2.4813
7 , 1	5	5.0576
7 , 3	7	4.2795
7 , 4	4	5.4467
7 , 7	3	2.8530
8 , 1	1	2.4726
8 , 3	4	2.0922
8 , 4	4	2.6628
8 , 7	2	2.8530
8 , 8	2	0.6657
9 , 1	1	0.4496
9 , 3	0	0.3804
9 , 4	0	0.4841
9 , 7	0	0.5187
9 , 8	0	0.2536
9 , 9	0	0.0173
10 , 1	0	0.4496
10 , 3	0	0.3804
10 , 4	0	0.4841
10 , 7	2	0.5187
10 , 8	0	0.2536
10 , 9	0	0.0461
10 , 10	0	0.0173
11 , 1	3	5.6196
11 , 3	4	4.7550
11 , 4	7	6.0519
11 , 7	7	6.4841
11 , 8	3	3.1700
11 , 9	2	0.5764
11 , 10	0	0.5764
11 , 11	3	3.5303
12 , 1	0	0.2248
12 , 3	1	0.1902
12 , 4	1	0.2421
12 , 7	0	0.2594
12 , 8	0	0.1268
12 , 9	0	0.0231
12 , 10	0	0.0231
12 , 11	0	0.2882
12 , 12	0	0.0029
13 , 1	8	5.1700
13 , 3	2	4.3746
13 , 4	2	5.5677
13 , 7	3	5.9654
13 , 8	1	2.9164
13 , 9	1	0.5303
13 , 10	2	0.5303
13 , 11	9	6.6282
13 , 12	0	0.2651
13 , 13	7	2.9827
14 , 1	0	1.2363
14 , 3	1	1.0461
14 , 4	3	1.3314

14 , 7	2	1.4265
14 , 8	0	0.6974
14 , 9	0	0.1268
14 , 10	0	0.1268
14 , 11	1	1.5850
14 , 12	0	0.0634
14 , 13	2	1.4582
14 , 14	0	0.1585
15 , 1	5	4.0461
15 , 3	3	3.4236
15 , 4	5	4.3573
15 , 7	6	4.6686
15 , 8	2	2.2824
15 , 9	0	0.4150
15 , 10	0	0.4150
15 , 11	8	5.1873
15 , 12	0	0.2075
15 , 13	1	4.7723
15 , 14	1	1.1412
15 , 15	2	1.8156
16 , 1	6	1.5735
16 , 3	1	1.3314
16 , 4	2	1.6945
16 , 7	1	1.8156
16 , 8	1	0.8876
16 , 9	0	0.1614
16 , 10	0	0.1614
16 , 11	0	2.0173
16 , 12	0	0.0807
16 , 13	1	1.8559
16 , 14	1	0.4438
16 , 15	1	1.4524
16 , 16	0	0.2622
